# CBKMR: A Copula-based Bayesian Kernel Machine Regression Framework for Optimal Marker Detection in Omics Data

**DOI:** 10.1101/2025.11.18.689140

**Published:** 2025-11-19

**Authors:** Anirban Chakraborty, Chloe Mattila, Debashis Ghosh, Brian Neelon, Souvik Seal

**Affiliations:** 1 Department of Public Health Sciences, Medical University of South Carolina, Charleston, South Carolina, USA; 2 Department of Biostatistics and Informatics, Colorado School of Public Health, University of Colorado Denver Anschutz Medical Campus, Aurora, Colorado, USA

**Keywords:** Bayesian variable selection, GKMR, Gaussian Copula, nearest neighbor GP approximation, Bulk omics, scRNA-seq

## Abstract

High-throughput bulk and single-cell omics technologies enable comprehensive molecular profiling, yet identifying compact, biologically interpretable marker sets that distinguish cell types, conditions, or disease states remains challenging. Standard pipelines rely on univariate differential expression tests, which ignore gene–gene dependencies and nonlinear effects, while multivariate machine-learning (ML) methods often lack principled feature selection and uncertainty quantification. The Bayesian kernel machine regression (BKMR) framework offers an appealing alternative because it (a) captures nonlinear gene–outcome relationships and higher-order interactions, and (b) enables automatic relevance determination (ARD) through sparsity-inducing priors. However, we show that the traditional latent Gaussian process (GP) formulation of BKMR is inadequate for discrete outcomes (e.g., cell-type labels), leading to biased inference and unstable variable selection. We propose a copula-based Bayesian kernel machine regression (CBKMR) model that uses outcome-appropriate discrete marginals while a Gaussian copula captures kernel-induced dependence across observations. To ensure scalability to modern single-cell datasets, we further introduce a nearest-neighbor GP-based variant, NNCBKMR, which reduces computational complexity from $𝒪\left(N^{3}\right)$ to nearly linear in N. Simulation studies show that CBKMR more accurately captures nonlinear effects and yields stronger marker-selection performance than BKMR and top ensemble ML methods (e.g., random forests, XGBoost). Applications to multiple scRNA-seq datasets demonstrate that CBKMR identifies concise marker panels that align closely with expert-annotated gene signatures while providingposterior uncertainty for principled decision-making.

## 1 Introduction

Over the past several years, high-throughput omics technologies, spanning both bulk and single-cell assays, have revolutionized our ability to quantify diverse molecular activities across complex biological systems^1,2^. These platforms enable comprehensive profiling of genes, proteins, and metabolites at multiple levels of biological resolution, opening new avenues for exploring cellular heterogeneity, tissue organization, developmental trajectories, and disease mechanisms. A parsimonious yet robust panel of biologically meaningful markers, i.e., molecular features that consistently characterize specific cell types, conditions, or disease states, is essential for clinical translation, as these can stratify patients, guide diagnostic and prognostic decisions, and inform therapeutic targeting^3–6^. Such a panel is also crucial for designing next-generation spatial transcriptomics (ST) and proteomics studies, where costs are substantial and informed gene or protein selection can markedly reduce assay burden^7^. Molecules often interact and exhibit nonlinear associations with biological outcomes (e.g., cell type identity or disease status), yet most analytical pipelines still rely on univariate differential expression (DE) tests^8–10^ for ad hoc marker selection, which overlook these complexities and fail to produce concise, predictive, and clinically translatable marker panels^11^.

Standard software packages for single-cell RNA sequencing (scRNA-seq) analysis, such as Seurat^12^ and Scanpy^13^, offer multiple options for detecting differentially expressed genes. These range from simple parametric and non-parametric pairwise tests, such as Welch’s t-test and the Wilcoxon rank-sum test, to more advanced model-based approaches, including hurdle model-based MAST^14^ and generalized linear models (GLMs) with negative binomial or Poisson likelihoods^15^. However, such independent, per-gene testing frameworks fail to account for joint dependencies among genes, making it difficult to systematically define a minimal subset of genes that captures maximal biological variation. In practice, detected genes are typically ranked by false discovery rate (FDR)–adjusted *p*-values, and an arbitrary cutoff is then applied to select the top-ranking genes as marker genes, a procedure that is inherently subjective and seldom reflects the true multivariate structure of the data. Furthermore, these approaches may overlook nonlinear relationships between gene expression and biological outcomes. To address these limitations, recent efforts have increasingly focused on predictive, multivariate modeling using machine learning (ML) and deep learning (DL) frameworks^16–21^. Some approaches leverage tree-based ensemble methods, such as random forests^22^ and XGBoost^23^, to model cell type or condition (e.g., one-vs-rest) as a binary response and gene-expression profiles as predictors. However, determining predictor significance through feature-importance thresholding remains ad hoc and non-trivial. Other approaches adopt optimization-based algorithms, including DL architectures, which often require extensive hyperparameter tuning and careful regularization. Therefore, there is a need for intuitive, automated, and uncertainty-aware marker-detection methods that can be readily adopted in biological and clinical research.

With the rapid surge of genome-wide association studies (GWAS) two decades ago, kernel machine regression (KMR) frameworks^24,25^ gained prominence as powerful tools for detecting nonlinear associations between individual or groups of SNPs and complex phenotypes^26–30^. Fundamentally, KMR extends traditional linear regression by relaxing the linearity assumption and introducing a semiparametric function, typically modeled as a realization from a Gaussian process (GP) with an appropriate kernel covariance function^31^. The kernel covariance structure captures the similarity between samples based on their feature expression profiles. Similar models have been recently used in scRNA-seq datasets, facilitating analogous association testing between genes and phenotypic outcomes such as cell type identity or disease status^32–34^. However, most of these studies emphasize the detection of nonlinearly differentially expressed genes, rather than predictive modeling aimed at identifying a compact, non-redundant set of informative markers. Consequently, the kernel covariance matrix is constructed using only a single feature or a small subset of features, implicitly assuming fixed or equal importance among them. In a conceptually related domain, namely, the identification of key exposures from complex environmental mixtures, the Bayesian KMR (BKMR) framework^35,36^ introduced a more flexible approach by leveraging the automatic relevance determination (ARD) kernel^37^, which allows predictors to assume varying levels of importance. By further placing a sparsity-inducing spike-and-slab prior^38^ on the inverse lengthscales, which govern the relevance of individual features, this formulation adaptively learns the relative importance of each predictor dimension within a unified modeling framework. The appeal of such an approach in our context is evident. One can envision a Bayesian logistic KMR^39^ with cell type or disease status as the outcome, where all genes are embedded within an ARD kernel covariance; the model automatically infers each gene’s relative importance and summarizes it through the posterior inclusion probability (PIP), providing an intuitive mechanism for selecting the final subset of informative markers.

Bobb et al. (2018)^40^ implemented a probit KMR model for binary outcomes with a similar variable selection strategy. More broadly, the KMR framework naturally extends to accommodate a wide class of exponential family outcomes, such as Gaussian, binomial, Poisson, and negative binomial, through the generalized KMR formulation^41^. Mou et al. (2025)^42^ recently introduced a Bayesian extension, generalized Bayesian KMR (GBKMR), which incorporates variable selection analogous to BKMR. These generalized KMR models share a common assumption: kernel-induced dependence is introduced through a latent Gaussian random effect, implicitly requiring that the joint dependence among responses be fully captured by correlations in a multivariate Gaussian latent space. While appropriate for continuous outcomes, this assumption becomes restrictive for discrete or otherwise non-Gaussian data. As Madsen et al. (2009)^43^ showed, for discrete outcomes the latent Gaussian correlation structure no longer *uniquely* determines the dependence among the observed responses. Consequently, latent GP–based mixed models (via link functions) often underrepresent or distort the true dependencies across observations, particularly when the discrete outcome distribution is skewed or overdispersed.

The above limitation motivates a copula-based construction^44^, which models the outcome distribution directly while using a flexible copula to encode kernel-driven dependence, thereby more accurately capturing complex, nonlinear, and non-Gaussian dependencies among discrete observations. We term the resulting framework *copula-based Bayesian kernel machine regression (CBKMR)*. Copula-based mixed models have a strong precedent in spatial statistics^45–48^, including the foundational work of Madsen et al. (2009). By modeling outcome distributions and dependence separately through a Gaussian copula, CBKMR provides a more faithful representation of complex associations and enables principled feature selection in high dimensions. In extensive evaluations, including settings with model misspecification, logistic CBKMR consistently outperforms GBKMR and leading ML ensemble methods such as random forests and XGBoost in detecting significant features. It further recovers compact, pathologist-validated marker sets in two independent scRNA-seq datasets. To ensure scalability to larger datasets, we incorporate a nearest-neighbor Gaussian process (NNGP) approximation^49,50^ within the copula formulation, yielding *NNCBKMR*, an efficient variant that retains CBKMR’s rigor while reducing computational complexity from $𝒪\left(N^{3}\right)$ to linear in N, where N denotes the total number of cells.

The remainder of the paper is organized as follows. Section 2.1–2.2 reviews GBKMR in the context of general exponential family distributions. Section 2.3 introduces the proposed CBKMR framework and details posterior inference. Section 2.5 presents the NNGP approximation for large datasets. Section 3 reports results from simulation studies. Findings from two scRNA-seq applications ar reported in Sec 4. We conclude in Section 5. An efficient GitHub package implemented in Rcpp is available.

## 2 Methods

### 2.1 Brief review of popular BKMR models

Conceptually related to the support vector machine (SVM)^51^, kernel-based Gaussian process (GP) methods have a longer history^52^ and have been widely applied in statistics and machine learning, including the Bayesian formulations^53,54,39,55,56^. For clarity, we refer to the framework introduced by Bobb et al. (2015) as BKMR throughout this manuscript. Since its introduction, BKMR has undergone substantial methodological extension and has been applied across diverse scientific domains. As a non-comprehensive overview of key developments, Crawford et al. (2018) introduced Bayesian approximate kernel regression, which approximates BKMR by replacing the full GP kernel with a random Fourier feature representation, yielding substantial computational gains. Liu et al. (2018)^57^ extended BKMR to accommodate both longitudinal and cross-sectional correlations. Teng et al. (2020)^58^ treated the kernel function as a random object, performing *automatic kernel selection* based on model evidence. Mutiso et al. (2024)^59^ adapted BKMR for negative binomial outcomes, illustrating its utility in modeling associations between social vulnerability and COVID-19 mortality in South Carolina. Smith et al. (2025)^60^ expanded BKMR to incorporate heteroskedastic error structures. Most recently, Mou et al. (2025)^42^ introduced a generalized BKMR framework, which serves as the foundation for our proposal.

### 2.2 Generalized Bayesian kernel machine regression

Suppose there are N cells, and we focus on a binary *one-vs-rest* classification for a given cell type. For i=1,…,N, let $Y_{i}$ denote the random variable representing the cell type label of the $i^{\mathrm{th}}$ cell, and let $y_{i}$ be its observed value. Thus, $Y_{i}$ is the underlying stochastic outcome, while $y_{i}$ is its realization in the data. For the $i^{\mathrm{th}}$ cell, we further consider two sets of covariates: $x_{i}\in R^{b}$, which is assumed to have a linear effect on $y_{i}$ (e.g., batch indicators), and $z_{i}\in R^{p}$, which is assumed to have a potentially nonlinear effect on $y_{i}$ (e.g., gene expression profiles). For a general discrete outcome, GBKMR assumes that $Y_{i}$ follows an exponential family distribution with density $f_{Y_{i}}\left(y_{i}|\theta_{i}\right)=exp\left\{\frac{y_{i}\theta_{i}-b\left(\theta_{i}\right)}{a\left(\phi_{i}\right)}+c\left(y_{i},\phi_{i}\right)\right\}$, where $E\left(Y_{i}\right)=\mu_{i}$, and the functions $a\left(\cdot \right)$, b(⋅), and c(⋅) are determined by the choice of the distribution for $Y_{i}$. In the binary case $Y_{i}\in \{0,\;1\}$, the model specializes to a Bernoulli likelihood with canonical logit link,

(2.1) $$\eta_{i}=g\left(\mu_{i}\right)=\beta_{0}+x_{i}^{⊤}\beta_{1}+h\left(z_{i}\right),\;i=1,\ldots ,N,$$

where g(⋅)=logit(⋅), $\beta_{0}$ is the intercept, and $\beta_{1}$ is the coefficient vector for the linear covariates $x_{i}$. The term $h\;:R^{p}\to R$ denotes a nonlinear function in a reproducing kernel Hilbert space (RKHS) ${ℋ}_{K}$ with a positive semidefinite reproducing kernel $K\;:R^{p}\times R^{p}\to R$. Under this assumption, the vector of evaluations $h={\left(h\left(z_{1}\right),\ldots ,h\left(z_{N}\right)\right)}^{⊤}$ follows an N-variate Gaussian distribution, $h\sim {𝒩}_{N}\left(0,\tau K_{r}\right)$ where ${𝒩}_{N}$ denotes an N-variate Gaussian distribution, τ>0 is a scale (roughness) parameter, and $K_{r}={\left(K\left(z_{i},z_{j}\right)\right)}_{i,j=1}^{N}$ is the kernel covariance matrix constructed based on K. GBKMR adopts an automatic relevance determination (ARD) Gaussian kernel^37^, which assigns a separate *inverse* length-scale (relevance) parameter $r_{m}\geq 0$ to each feature dimension:

(2.2) $$K_{r,ij}=exp\left(-\sum_{m=1}^{p}\;r_{l}{\left(z_{im}-z_{jm}\right)}^{2}\right),$$

where a large $r_{m}$ indicates greater relevance of the feature. The parameter set for (2.1)–(2.2) is $\theta ={\left(\beta_{0},\beta_{1}^{⊤},\tau ,r_{1},\ldots ,r_{p}\right)}^{⊤}$, The Markov chain Monte Carlo (MCMC) procedure for posterior inference on θ based on an iterative reweighted least squares (IRLS) approach is provided in Mou et al. (2025)^42^.

### 2.3 Copula-based Bayesian kernel machine regression (CBKMR)

To construct the copula-based BKMR (CBKMR) model, we retain the previous notation and let $F_{i}(\cdot )$ denote the cumulative distribution function (CDF) of $Y_{i}$. As in (2.1), we adopt a logit link to specify the marginal mean structure, modeling *only* the linear effects,

(2.3) $$\eta_{i}=g\left(\mu_{i}\right)=\beta_{0}+x_{i}^{⊤}\beta_{1},\;i=1,2,\ldots ,N.$$

We focus on a Bernoulli outcome for clarity; however, the model is directly applicable to other discrete distributions through a suitable choice of link function g(⋅). We assume a Gaussian copula constructed from the marginal CDFs: ${\left\{F_{i}\left(y_{i}\right)\right\}}_{i=1}^{N}$ to characterize dependence among the observations $y_{i}$^43^, resulting

(2.4) $$C\left(y;\Sigma_{r}\right)=\Phi_{N,\Sigma_{r}}\left(\Phi^{-1}\left(F_{1}\left(y_{1}\right)\right),\ldots ,\Phi^{-1}\left(F_{N}\left(y_{N}\right)\right)\right),$$

where $C\;:R^{N}\to R$ denotes the Gaussian copula CDF, $\Phi_{N,\Sigma_{r}}(\cdot )$ is the CDF of an N-variate normal distribution with correlation matrix $\Sigma_{r}$, and Φ(⋅) is the CDF of a univariate standard normal distribution. We parameterize the correlation matrix as

(2.5) $$\Sigma_{r}=\tau K_{r}+(1-\tau )I_{N},$$

where 0<τ≤1 and $K_{r}$ is the ARD Gaussian kernel from (2.2). The shrinkage parameter τ controls the contribution of nonlinear dependence: when τ≈1, CBKMR behaves like a fully kernel-dependent model, whereas smaller τ values shrink $\Sigma_{r}$ toward the identity, allowing observations to become (partially or fully) independent. This decomposition stabilizes inference in weak-signal settings and mitigates over-smoothing from the copula kernel.

Our next objective is to derive a density function corresponding to C(⋅). When $F_{i}$’s are continuous, the corresponding density is uniquely defined on $R^{N}$ and can be easily derived using Sklar’s theorem^61^,

(2.6) $$f\left(y|\tau ,\Sigma_{r}\right)=c\left(y;\Sigma_{r}\right)\prod_{i=1}^{N}f_{i}\left(y_{i}\right),\;c\left(y;\Sigma_{r}\right)=\frac{1}{{\left|\Sigma_{r}\right|}^{1/2}}exp\left(-\frac{1}{2}{\left[\Phi^{-1}(F(y))\right]}^{⊤}\left(\Sigma_{r}^{-1}-I_{n}\right)\left[\Phi^{-1}(F(y))\right]\right),$$

where $F(y)={\left(F_{1}\left(y_{1}\right),\ldots ,F_{n}\left(y_{N}\right)\right)}^{⊤}$ and $c\left(y;\Sigma_{r}\right)$ is the Gaussian copula density. However, because the marginals $F_{i}$ are step functions in our setting, the copula C does not possess continuous support on $R^{N}$, and Sklar’s theorem cannot be applied via differentiation to obtain a valid likelihood^45^. In principle, the likelihood could be recovered through an inclusion–exclusion expansion, but this requires summing over $2^{N}$ terms^43^, making it computationally infeasible even for moderate N. To overcome this limitation, we adopt a distributional transform (DT) approximation^62^, which has been shown to yield accurate Gaussian copula likelihoods for discrete marginals. For any $W_{i}\sim Unif(0,\;1)$ independent of $Y_{i}$, the DT of $Y_{i}$ is defined as

$$F_{i}\left(W_{i},Y_{i}\right)=\left(1-W_{i}\right)F_{i}\left(Y_{i}^{-}\right)+W_{i}F_{i}\left(Y_{i}\right),$$

where $F_{i}\left(Y_{i}^{-}\right)=P\left(Y_{i}\leq y_{i}-1\right)$. Following Hughes et al. (2015)^45^, we replace $F_{i}\left(y_{i}\right)$ by its conditional expectation under the DT,

(2.7) $$u_{i}^{\prime }=E\left[F_{i}\left(W_{i},Y_{i}\right)|Y_{i}=y_{i}\right]=\frac{F_{i}\left(y_{i}\right)\;+\;F_{i}\left(y_{i}\;-\;1\right)}{2}.$$

Substituting $u_{i}^{\prime }$ into the copula representation in (2.6) yields the approximate CBKMR likelihood:

(2.8) $$f_{DT}\left(y|\tau ,r,\beta_{0},\beta_{1}\right)=c\left(u^{\prime };\Sigma_{r}\right)\prod_{i=1}^{N}f_{i}\left(y_{i}\right),$$

where $u^{\prime }={\left(u_{1}^{\prime },\ldots ,u_{N}^{\prime }\right)}^{⊤}$, and $f_{i}(\cdot )=f_{Y_{i}}(\cdot )$ denotes the Bernoulli probability mass function (pmf).

Such a copula-based BKMR formulation offers a key advantage over GBKMR: it is invariant to the choice of the marginal distribution. For any outcome type, we only specify a suitable link function g(⋅) to relate $\mu_{i}$ to $x_{i}$ and define the marginals $F_{i}(\cdot )$, while the dependence structure remains unchanged. In contrast, GBKMR embeds the kernel-induced dependence *inside* the link function, which can be restrictive or unstable for discrete responses due to non-Gaussian dependence among observations^43,45^. By separating marginal and dependence modeling, CBKMR provides more robust inference for discrete outcomes, as demonstrated in Section 3. We next describe variable selection and the MCMC algorithm used to estimate the parameter vector $\theta ={\left(\beta_{0},\beta_{1}^{⊤},r_{1},\ldots ,r_{p},\tau \right)}^{⊤}$.

### 2.4 Sparsity prior and posterior inference

To devise an MCMC algorithm for θ we first assign a Beta prior distributions on the shrinkage parameter τ and Gaussian prior on the parameters $\beta_{0}$ and $\beta_{1}$,

(2.9) $$\tau \sim Beta\left(a_{\tau },b_{\tau }\right),\;\beta_{0}\sim 𝒩\left(0,b^{2}\right),\;\beta_{1}\sim {𝒩}_{b}(0,\Omega ),$$

where $a_{\tau }$, $b_{\tau }$, $b^{2}$ and Ω are prespecified hyperparameters. To induce sparsity within the ARD kernel and enable effective variable selection, the prior on the relevance parameters $\left\{r_{m}\right\}$ must place positive mass at zero, allowing weakly informative features to be *switched off*. Following Bobb et al. (2015)^36^, we consider a spike-and-slab prior^38^ as

(2.10) $$r_{m}|\delta_{m}\sim \delta_{m}g_{1}\left(r_{m};r_{a},r_{b}\right)+\left(1-\delta_{m}\right)P_{0},\;\delta_{m}\sim Bernoulli\left(\pi \right),\;\pi \sim Beta\left(a_{\pi },b_{\pi }\right),\;m=1,\ldots ,p,$$

where $g_{1}\left(r_{m};r_{a},r_{b}\right)$ is the density of a $Uniform\left(r_{a},r_{b}\right)$ distribution, $P_{0}$ is a degenerate mass at 0, and $a_{\pi }$ and $b_{\pi }$ are the parameters of the Beta distribution. The full posterior distribution of the set of augmented parameters $\tilde{\theta }=\left(\theta ,\delta_{1},\ldots ,\delta_{v}\right)$ can be expressed as,

(2.11) $$p\left(r,\delta ,\beta_{0},\beta_{1},\tau |y\right)\propto c\left(u^{\prime };\Sigma_{r}\right)[\prod_{i=1}^{N}\;f_{i}\left(y_{i}\right)][\prod_{i=1}^{p}\;p\left(r_{i}|\delta_{i}\right)]p(\delta )p\left(\beta_{0}\right)p\left(\beta_{1}\right)p(\tau )$$

where $c\left(u^{\prime },\Sigma_{r}\right)$ is the copula density in equation (2.6), p(⋅)’s denote the prior densities of the corresponding parameters. The MCMC algorithm proceeds in three steps - (i) update of $\beta_{0}$ and $\beta_{1}$, (ii) update of the relevance and inclusion parameters ($r_{i},\delta_{i}$), and (iii) update of the shrinkage parameter τ. Unlike standard logistic GLMs, where Pólya–Gamma data augmentation^63,64^ allows for direct sampling of $\beta_{0}$ and $\beta_{1}$, the presence of the copula density c(⋅) in our model precludes standard Gibbs updates. Consequently, we implement a Metropolis-Hastings (MH) algorithm to sample these parameters, leveraging a modified Pólya–Gamma augmentation to facilitate efficient mixing. Update of τ is obtained through a random walk MH-based algorithm. Finally, we follow Bobb et. al. (2015)^36^ to perform a reversible jump Markov chain Monte Carlo (RJMCMC) algorithm for updating r and δ. We elaborate the steps in Algorithm 1. Once MCMC is completed and burn-ins are discarded, we calculate posterior inclusion probabilities (PIPs) for the $m^{\mathrm{th}}$ covariate in $z_{i}$’s as ${PIP}_{m}=\frac{1}{B}\sum_{t=1}^{B}\delta_{m}^{\left(t\right)}$. For a given threshold *thres* (say, 0.75), the $m^{\mathrm{th}}$ covariate is selected if ${PIP}_{m}\geq \;thres$. A higher value of *thres* leads to a conservative selection of variables.

#### 2.4.1 Prediction at new observation

After performing inference on existing observations, it is generally of interest to predict the unobserved response for a new sample with known covariates. Proposition 1 outlines the approximate probability mass function of the unobserved response for the corresponding known covariates.

##### Proposition 1.

*Suppose* $X_{N}={\left(x_{1}^{⊤},\ldots ,x_{N}\right)}^{⊤},\;Z_{N}={\left(z_{1}^{⊤},\ldots ,z_{N}^{⊤}\right)}^{⊤}$ *and* $y=\left(y_{1},\ldots ,y_{N}\right)$ *denote the observed covariates and responses for* N *individuals. The posterior distribution of random variable* $Y_{N+1}$ *for an unobserved response with known covariates* $x_{N+1}$ *and* $z_{n+1}$ *for given value of parameters follow a discrete distribution with its probability mass function expressed as*

**Algorithm 1:** MCMC algorithm for CBKMR

1:	**Output.** $\beta_{1}^{\left(1:B\right)}$, where $\beta_{1}^{\left(t\right)}={\left(\beta_{11}^{\left(t\right)},\ldots ,\beta_{1b}^{\left(t\right)}\right)}^{T}$, $\delta^{\left(1:B\right)}$, where $\delta^{\left(t\right)}={\left(\delta_{1}^{\left(t\right)},\ldots ,\delta_{p}^{\left(t\right)}\right)}^{T}$, $r^{\left(1:B\right)}$, where $r^{\left(t\right)}={\left(r_{1}^{\left(t\right)},\ldots ,r_{p}^{\left(t\right)}\right)}^{⊤}$, τ, t=1,…,B.	
2:	Initialize $\beta_{0}$, $\beta_{1}$, δ, r, τ.	
3:	**for** t=1,…,T **do**	
4:	Sample $\beta_{0}$, $\beta_{1}$	
5:	Accept/ reject $\beta_{0}$ with the MH acceptance probability	$𝒪\left(N^{3}\right)$ flops
$\alpha (\beta_{0}^{\left(t+1\right)},\beta_{0}^{\left(t\right)})=min\left\{1,\frac{c\left(u^{\prime }\left(\beta_{0}^{\left(t+1\right)}\right);\Sigma_{r}\right)\prod_{i=1}^{N}\;f_{i}^{\beta_{0}^{\left(t+1\right)}}\left(y_{i}\right)p\left(\beta_{0}^{\left(t+1\right)}\right)q\left(\beta_{0}^{\left(t\right)}|\beta_{0}^{\left(t+1\right)}\right)}{c\left(u^{\prime }\left(\beta_{0}^{\left(t\right)}\right);\Sigma_{r}\right)\prod_{i=1}^{N}\;f_{i}^{\beta_{0}^{\left(t\right)}}\left(y_{i}\right)p\left(\beta_{0}^{\left(t+1\right)}\right)q\left(\beta_{0}^{\left(t+1\right)}|\beta_{0}^{\left(t\right)}\right)}\right\},$		
6:	Perform a modified Pólya-Gamma-based MH algorithm step for $\beta_{1}$.	
7:		
8:	**Sample** (δ,r)	
9:	Randomly choose two moves from below:	
10:	i. Randomly choose k∈{1,…,p}. If $\delta_{k}=1$, set $\left(\delta_{k},r_{k}\right)=(0,\;0)$; else set $\delta_{k}=1$, and propose $r_{k}^{\left(t+1\right)}∼q_{r}(\cdot |r_{k}^{\left(t\right)})$, from the proposal $q_{r}$.	
11:	ii. Among the k s such that $\delta_{k}=1$, propose $r_{k}^{\left(t+1\right)}∼q_{r}(\cdot |r_{k}^{\left(t\right)})$, from the proposal $q_{r}$.	
12:	Accept/ reject $r^{\left(t+1\right)}$ using MH algorithm (similar to step 5, with $q(\beta^{\left(t+1\right)}|\beta^{\left(t\right)})$ being replaced by $\left.q_{r}\left(r^{\left(t+1\right)}|r^{\left(t\right)}\right)\right)$.	$𝒪\left(N^{3}\right)$ flops
13:		
14:	**Sample** τ	
15:	Propose a new sample $\tau^{\left(t+1\right)}∼q_{\tau }(\cdot |\tau^{\left(t\right)})$ and accept/ reject using MH algorithm (similar to step 5, with $q\left(\beta^{\left(t+1\right)}|\beta^{\left(t\right)}\right)$ being replaced by $\left.q_{\tau }\left(\tau^{\left(t+1\right)}|\tau^{\left(t\right)}\right)\right)$.	$𝒪\left(N^{3}\right)$ flops
16:	**end for**	
17:	Calculate ${PIP}_{m}=\frac{1}{B}\sum_{t=1}^{B}\delta_{m}^{\left(t\right)}$. Select $z_{m}$, if ${PIP}_{m}\geq$ thres, m=1,…,p.	


(2.12) $$f_{Y_{N+1}}\left(y|r,\beta_{0},\beta_{1},y\right)=h\left(r,\beta_{0},\beta_{1},y\right)c\left(u_{N+1}^{\prime };\Sigma_{r}\right)f_{N+1}\left(y|\beta_{0},\beta_{1}\right),$$

*where* $f_{N+1}\left(\cdot |\beta_{0},\beta_{1}\right)$ *denotes the marginal distribution of* $Y_{N+1},c\left(u_{N+1}^{\prime },\Sigma_{r}\right)=\tau K_{r}+(1-\tau )I_{N+1}$ *is the* $N+1\times N+1,\;where\;K_{N+1}$ *is the* N+1×N+1 *Gaussian covariance matrix, and* h(⋅) *is a normalizing constant, such that*, $h\left(r,\beta_{0},\beta_{1},y\right)=\sum_{y}{\left[c\left(u_{N+1}^{\prime };\Sigma_{r}\right)f_{N+1}\left(y|\beta_{0},\beta_{1}\right)\right]}^{-1}$.

When $Y_{i}\in \{0,\;1\}$, we can easily compute the pmf using Proposition 1. Furthermore, by varying the values of the parameters, we can obtain a continuous distribution over the success probabilities.

### 2.5 Nearest neighbor CBKMR (NNCBKMR) for large datasets

The MCMC algorithm in Section 2.4 involves computation of the copula density c(⋅) in equation (2.8). This incurs $𝒪\left(N^{2}\right)$ memory and $𝒪\left(N^{3}\right)$ time at every iteration due to matrix-inversion of $\Sigma_{r}$^31^. Consequently, CBKMR is prohibitive to implement even for a moderately large N (e.g., ~ 10,000). Here, we propose a scalable CBKMR approach by adopting a nearest neighbor Gaussian process (NNGP)-based sparse Cholesky decomposition approximation^65,49,66^. To elucidate our approach, we temporarily suppress all the notations of CBKMR, and assume that a random vector $x∼{𝒩}_{N}(0,\Sigma ={\left({UU}^{⊤}\right)}^{-1})$, where U is the Cholesky factor of $\Sigma^{-1}$. Motivated by the decomposition of any N-variate density into N univariate conditional densities $\;f(x)=\prod_{i=1}^{N}f\left(x_{i}|x_{1:i-1}\right)$, Vecchia et. al. (1988)^65^ proposed an approximate density $\overset{ˆ}{f}(x)=\prod_{i=1}^{N}f\left(x_{i}|x_{Ne(i)}\right)$, where Ne(i)⊂{1,…,i} is a subset of suitably chosen nearest neighbors (NNs) for the indices i. When applied to a N-variate Gaussian distribution, this yields a lower triangular sparse Cholesky factor $U_{𝒮}$ of $\Sigma^{-1}$, such that $U_{𝒮;i,j}=0$ whenever j∉Ne(i)^49,66^. The sparsity structure 𝒮 contains the information of neighbors Ne(i)’s. By fixing the maximum neighbor size ${max}_{i}\;|Ne(i)|=k$, computation of the Gaussian likelihood using $U_{𝒮}$ requires only 𝒪(Nk) memory and $𝒪\left(Nk^{3}\right)$ time. While k=N-1 gives back the exact decomposition, in practice almost accurate approximation can be achieved with k∼40 even for a very large N ^50^. Since the copula density c(⋅) imitates a Gaussian likelihood, this sparse decomposition can be implemented to compute c(⋅).

#### Definition 1.

Suppose, $U_{r,𝒮}$ denotes a lower triangular sparse Cholesky factor of $\Sigma_{r}^{-1}$ in (2.8) for a given sparsity structure 𝒮, such that ${max}_{i}\;|Ne(i)|=k$. Then the NN approximated density of y *is given by*

(2.13) $$f_{NNDT}\left(y|\tau ,r,\beta_{0},\beta_{1},U_{𝒮}\right)=c(u^{\prime };{\left(U_{r,𝒮}U_{r,𝒮}^{⊤}\right)}^{-1})\prod_{i=1}^{N}f_{i}\left(y_{i}\right),$$

In addition to a guaranteed fast computation, $f_{NNDT}$ is also approximately optimal in terms of KullbackLeibler (KL) divergence minimization.

#### Lemma 1.

*Suppose* y *is a* N-*variate vector that follows a Gaussian copula* $C\left(y;\Sigma_{r}\right)$ *with corresponding density* $c(\cdot ),\;continuous\;marginals\;\left\{F_{i}\right\}$ *and density* $\left\{f_{i}\right\}.\;In\;addition,\;assume\;U_{r,𝒮}$ *is the sparse Cholesky decomposition of* $\Sigma_{r}^{-1}$ *for a given sparsity structure. Then*

- The nonzero entries in the $i^{th}$ column of $U_{r,𝒮}$ are given by $L_{r;Ne(i),i}=\frac{\Sigma_{r;Ne(i),Ne(i)}^{-1}e_{1}}{\sqrt{e_{1}^{⊤}\Sigma_{r;Ne(i),Ne(i)}^{-1}e_{1}}}$,
- $U_{r,𝒮}=\underset{A_{𝒮}\in R^{N\times N}:A_{𝒮}>0,A_{𝒮,ij}=0\forall j>i,j\notin Ne(i)}{argmin}KL\left(f_{DT}\left(y|\tau ,r,\beta_{0},\beta_{1}\right)||f_{NNDT}\left(y|\tau ,r,\beta_{0},\beta_{1},A_{𝒮}\right)\right)$,

*where* $e_{1}\in R^{|Ne(i)|\times i}$ *has the first entry equal to 1 and all other entries equal to 0.* $\;A_{𝒮}>0$ *implies that* $A_{𝒮}$ *is positive definite.*

To maintain clarity of the discussion, we postpone the proof to Section A. The lemma 1 suggests that the NN approximated $f_{NNDT}\left(y|\tau ,r,\beta_{0},\beta_{1},L_{r,𝒮}\right)$ is an optimal approximation to $f_{DT}\left(y|\tau ,r,\beta_{0},\beta_{1}\right)$, in Eq. 2.8.

To compute $U_{r,𝒮}$, we first perform a *maxmin* permutation^67^ of the observations and use a k-d tree-based algorithm from GpGp R package to find the nearest neighbors from the relevance-transformed inputs $\tilde{Z}=Z\;diag\left(r^{1/2}\right)$. The resulting $f_{NNDT}$ simply replaces $f_{DT}$ in Section 2.4 for the MCMC steps of NNCBKMR. For clarity, we provide a pseudocode in algorithm 2.

While NNCBKMR is computationally efficient, it still requires a nearest neighbor search at each iteration following the update of r. Theoretically, this operation incurs a time complexity of 𝒪(pNlogN)^68^. However, in many practical applications, the vector r exhibits significant sparsity, with only a small subset of non-zero entries $r_{m}$. Consequently, the effective computational cost of the nearest neighbor search remains tractable.

**Algorithm 2:** MCMC algorithm for NNCBKMR

1:	**Output.** $\beta_{1}^{\left(1:B\right)}$, where $\beta_{1}^{\left(t\right)}={\left(\beta_{11}^{\left(t\right)},\ldots ,\beta_{1b}^{\left(t\right)}\right)}^{T}$, $\delta^{\left(1:B\right)}$, where $\delta^{\left(t\right)}={\left(\delta_{1}^{\left(t\right)},\ldots ,\delta_{p}^{\left(t\right)}\right)}^{T}$, $r^{\left(1:B\right)}$, where $r^{\left(t\right)}={\left(r_{1}^{\left(t\right)},\ldots ,r_{p}^{\left(t\right)}\right)}^{⊤}$, τ, t=1,…,B.	
2:	Initialize $\beta_{0}$, $\beta_{1}$, δ, r, τ.	
3:	Permuteorder=maxmin(Z), y=y[order], X=X[order,], Z=Z[order,].	
4:	$k=\underset{i}{max}\;|Ne(i)|$. Neighbor finder ${NN}_{k}$. Find $𝒮={ZNN}_{k}\left(diag\left(r^{1/2}\right)\right)$.	
5:	**for** t=1,…,T **do**	
6:	**Sample** $\beta_{0}$, $\beta_{1}$	
7:	Accept/ reject $\beta_{0},\beta_{1}$ - same as algorithm 1, with $f_{DT}$ replaced by $f_{NNDT}$.	$𝒪\left(Nk^{3}\right)$ flops
8:		
9:	**Sample** (δ,r)	
10:	Same RJMCMC and MH step as in algorithm 1.	
11:	For a new proposed $r^{\prime }$, calculate ${𝒮}^{\prime }={ZNN}_{k}\left(diag\left(r^{\prime 1/2}\right)\right)$.	𝒪(pNlogN) flops
12:	Same step as in algorithm $1,f_{DT}$ replaced by $f_{NNDT}$.	$𝒪\left(Nk^{3}\right)$ flops
13:	If $r^{\prime }$ is accepted, $𝒮={𝒮}^{\prime }$.	
14:		
15:	**Sample** τ	
16:	Same as in algorithm 1, with $f_{DT}$ replaced by $f_{NNDT}$.	$𝒪\left(Nk^{3}\right)$ flops.
17:	**end for**	
18:	Calculate ${PIP}_{m}=\frac{1}{B}\sum_{t=1}^{B}\;\delta_{m}^{\left(t\right)}$. Select $z_{m}$, if ${PIP}_{m}\geq$ thres, m=1,…,p.	

## 3 Simulation experiments

Our simulation experiments share two common parameters N and p. To generate the design matrix $Z={\left(z\cdot 1,\ldots ,z_{\cdot }\right)}^{⊤}$, where $z_{\cdot m}={\left(z_{1m},\ldots ,z_{Nm}\right)}^{⊤}$, we generate $z_{\cdot 1}$ from an N-variate standard Gaussian distribution. For m=2,…,p, we draw an N-variate standard Gaussian vector and set $z_{m}$ to be the residual from regressing this vector on $z_{\cdot 1},\ldots ,z_{\cdot m-1}$. This yields mutually orthogonal covariate vectors while preserving Gaussianity. We fix $\beta_{0}=0$ and $\beta_{1}=0$, i.e., no intercept or linear covariates x. To assess robustness to different dependence structures, we consider three simulation designs, each defined by a distinct mechanism linking predictors to the outcome. In all settings, data are generated from the GBKMR model in Eq. 2.1, and thus CBKMR is evaluated under *model misspecification*.

### Design 1.

We first simulate $\eta =\left(\eta_{1},\ldots ,\eta_{N}\right)$ from a N-variate Gaussian distribution,

$$\eta =h∼{𝒩}_{N}\left(0,\sigma_{r}^{2}K_{r}\right),\;K_{r,i,j}=\exp\left(-\sum_{d=1}^{p}\;r_{d}{\left(z_{id}-z_{jd}\right)}^{2}\right),$$

where $\sigma_{r}^{2}$ is the marginal variance and $r={\left(r_{1},\ldots ,r_{p}\right)}^{⊤}$ is the p-variate relevance vector. We consider only the first 3 variables to be significant, i.e., $r_{d}=1$, for d≤3, and $r_{d}=0\;\forall d>3$. By varying the values of r, $\sigma_{r}^{2}$, N and p we create several simulation scenarios under this setting. The marginal kernel variance $\sigma_{r}^{2}$ effectively controls how much of the relevances in r will be reflected in the response. A smaller value of $\sigma_{r}^{2}$ would eliminate any effect of r. As $\sigma_{r}^{2}$ increases, the effect of r becomes more prominent.

### Design 2.

We simulate $\eta_{i}$’s through an additive model similar to Mou et al. (2025),

$$\eta_{i}=\left(1.5z_{i1}+0.5z_{i2}^{2}+2sin\left(1.5z_{i3}\right)\right),\;i=1,\ldots ,N,$$

Notice that we consider only the first 3 components of each $z_{i}^{⊤}$ as significant.

### Design 3.

Similar to Design 2, we consider another additive model but with more significant covariates,

$$\eta_{i}=2\left(1.5z_{i1}+0.75z_{i2}^{2}+2sin\left(1.5z_{i3}\right)+\frac{1}{z_{i4}^{2}}+3sin\left(z_{i5}^{3}\right)+exp\left(-sin\left(z_{i6}^{2}\right)\right)\right),\;i=1,\ldots ,N,$$

We consider first 6 components of $z_{i}$’s as significant.

For all three designs and corresponding scenarios, we generate $y_{i}$’s from a Bernoulli distribution: $y_{i}∼Bernoulli\left(\mu_{i}\right)$, $\mu_{i}=\frac{e^{\eta_{i}}}{1+e^{\eta_{i}}}$, i=1,…,N. We evaluate four methods - CBKMR, GBKMR, random forests (RF), and XGBoost (XGB), using the area under the precision-recall curve (AUPRC) averaged over 50 replications as a metric for comparison. For CBKMR and GBKMR, we generated precision-recall curves by varying the PIP threshold over the interval [0, 1]. Since RF and XGBoost do not provide direct inclusion probabilities, we utilized their variable importance scores to construct analogous curves. Specifically, we ranked all variables by their importance scores (similar to Pullin et. al. (2024)^11^) and calculated precision and recall by varying the number of top-ranked variables selected, ranging from the true sparsity level up to the total number of predictors p.

**Figure 2:**
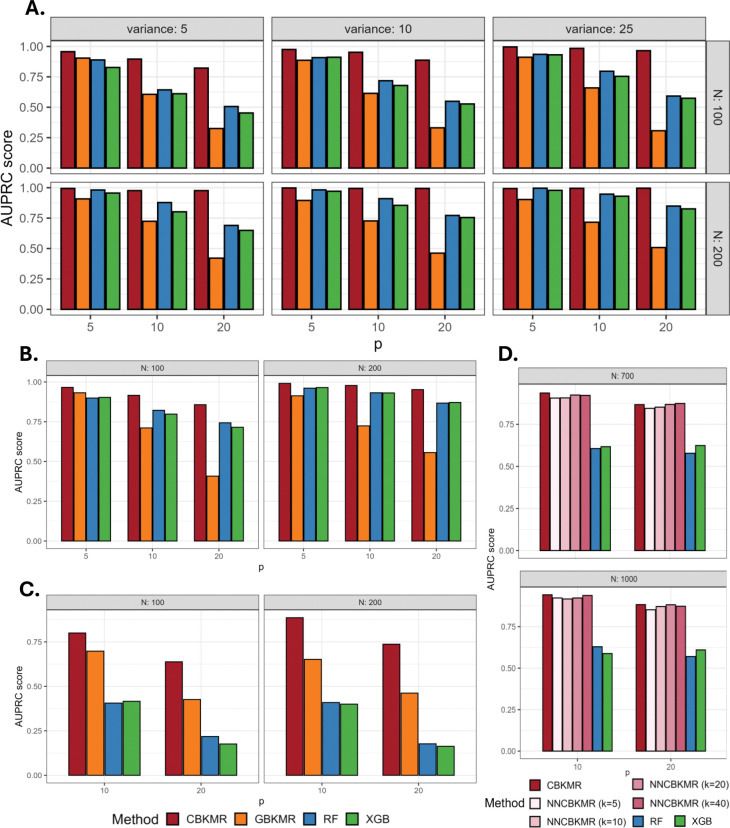
AUPRC score comparisons for different designs in Section 3. **A.** Design 1 as the variance ($\sigma_{r}^{2}$) increases. **B.** Design 2 with two values of N. **C.** Design 3 with two values of N. **D.** Design 3 with two larger values of N (700 and 1, 000), to evaluate NNCBKMR for varying sizes of the nearest neighbor set (k). GBKMR is ignored in this case for excessive runtime.

Figure 2 presents the AUPRC scores across all simulation designs. CBKMR consistently outperforms the competing methods, in some instances achieving a multi-fold improvement in accuracy over GBKMR (e.g., Figure 2A: N=100, p=20, $\sigma_{r}^{2}=10$). Notably, CBKMR substantially outperforms GBKMR in Design 1 (Figure 2A), despite the fact that the outcomes were simulated directly from a logistic GBKMR model with an ARD kernel. This observation aligns with findings by Madsen et al. (2009)^43^ and Hughes et al. (2015)^45^, which show that modeling dependence solely through the marginal link function is often inadequate for discrete outcomes. Furthermore, Figure 2C shows a substantial performance gain over ensemble ML models. While both RF and XGBoost perform poorly—failing to prioritize the true non-zero effects in their importance score rankings, particularly as the proportion of relevant variables increases—CBKMR maintains high AUPRC, demonstrating robust recovery of true signals with strong specificity.

### 3.1 Efficiency of NNCBKMR in comparison to CBKMR

To compare the accuracy and efficiency of NNCBKMR in large datasets, we consider 4 settings from Design 3 with N between (700, 1000) and p between (10, 20). For completeness, we also add RF and XGB to our comparison. We avoid running GBKMR in this setting due to its prohibitive computational cost. From 2D, it is evident that even with k as low as 20, NNCBKMR achieves a very close performance to the standard CBKMR. Furthermore, as seen in table 1, NNCBKMR with N=700 is almost 4 times faster for k=20 and 2.5 times faster for k=40 as compared to the standard CBKMR. When N=1000, NNCBKMR accelerates to 7.6 times faster with k=20 and 4.7 times faster with k=40. Such a computational gain will become further evident as N increases, underscoring the practical utility of NNCBKMR in large datasets.

## 4 Real data analysis

### 4.1 Analysis of Type 2 diabetes islet-cell data

Lawlor et al. (2017) generated single-cell transcriptomes (26,616 genes) from 638 human pancreatic islet cells collected from both non-diabetic and type 2 diabetic (T2D) donors^69^. Their analysis identified distinct transcriptional signatures for the major endocrine islet cell types (e.g., alpha, beta, delta, and gamma/PP cells) and revealed rare cellular states associated with islet dysfunction. Importantly, the dataset highlighted cell-type–specific expression changes in T2D that were not detectable in bulk islet profiling. In their downstream analyses, the authors relied on a curated set of canonical marker genes to annotate cell types, including *INS* (beta), *GCG* (alpha), *SST* (delta), *PPY* (gamma/PP), *PRSS1* (acinar), *COL1A1* (stellate), and *KRT19* (ductal). After minimal quality-control filtering, we retained 604 cells with the following highly skewed distribution of cell-type labels: alpha (238), beta (252), acinar (23), delta (20), ductal (23), gamma/PP (17), stellate (19), and other (12). We first performed univariate DE analysis for the seven cell types using a *cell-type–versus–rest* approach with *scran*’s Wilcoxon rank-sum test^70^, yielding 164 genes at an FDR threshold of 0.05. Notably, *SST* and *PPY* were absent from this set, highlighting limitations of univariate DE methods. We then removed highly correlated genes (pairwise correlation > 0.75) using *caret*’s findCorrelation function^71^, resulting in a final set of 132 genes that included all seven canonical markers. We evaluated CBKMR by examining whether it could successfully recover the canonical marker for every cell type.

**Figure 3:**
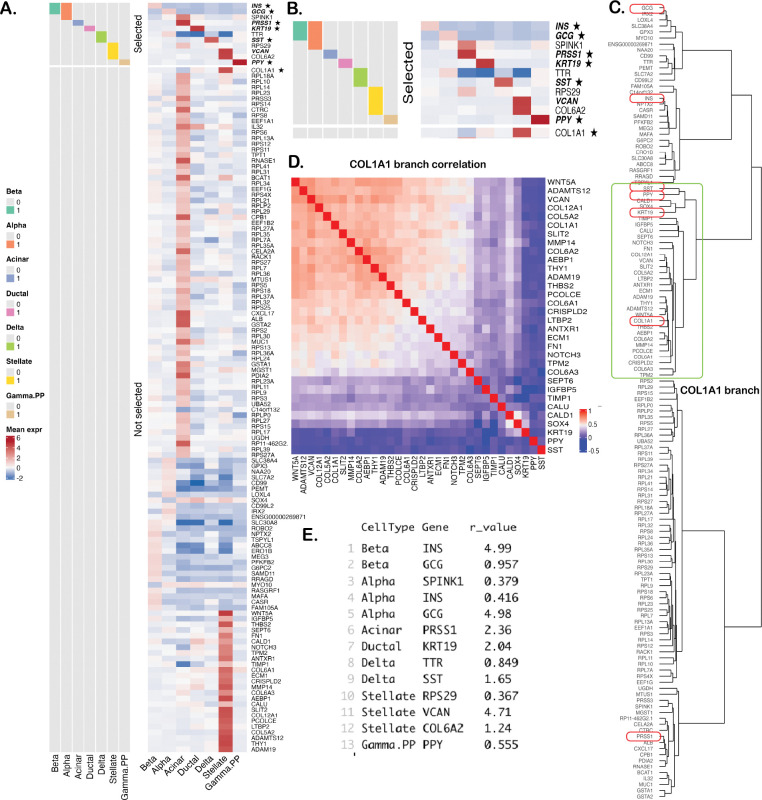
**A.** Heatmap of the average expression of 132 genes across seven cell types. Pathologist-annotated markers for each cell type are indicated with an asterisk (*). CBKMR-selected genes are shown for each cell type (0 = not selected, 1 = selected), with the *top* feature highlighted in bold. **B.** Zoomed view of the CBKMR-selected genes. **C.** Hierarchical clustering of genes; red boxes denote the markers, and the green box highlights the branch containing *COL1A1*. **D.** Correlation heatmap of genes within the *COL1A1* branch. **E.** Estimated inverse lengthscales (relevance) for the selected genes.

CBKMR detected 16 genes with PIP greater than 0.95 (most equal to 1), of which 13 also had estimated inverse lengthscales $r_{m}>0.25$. In Figs. 3A and 3B, we display the mean expression of all genes and highlight these 13 CBKMR-selected genes for each cell type. Notably, CBKMR identifies six out of seven canonical markers as top-ranked features, with the exception of *COL1A1* for stellate cells. As shown in Fig. 3C, the genes exhibit strong clustering, a consequence of the relatively permissive correlation threshold of 0.75 used during preprocessing. In Fig. 3D, we observe that most genes in the *COL1A1* cluster are highly correlated with it. In particular, *VCAN* and *COL6A2*—both selected by CBKMR for stellate cells—show substantial correlation with *COL1A1* (approximately 0.65) and have large inverse lengthscales of 4.71 and 1.24, respectively (Fig. 3E). This behavior likely reflects the fact that CBKMR does not explicitly model correlations among covariates and may tend to select a representative gene from a correlated block, an effect amplified by class imbalance (19 stellate cells vs. 585 other cells). Interestingly, *VCAN* and *COL1A1* have also been reported together as prognostic biomarkers in gastric cancer^72^. CBKMR’s strong overall performance underscores its utility for complex real datasets in which genes exhibit substantial correlation. While the correlation threshold could be tightened to yield a smaller candidate gene set, we intentionally used this more extreme setting to evaluate performance in a highly correlated scenario. For random forests and XGBoost, feature selection based on variable importance is inherently ad hoc; therefore, we focus on the top-ranked gene identified by each method. Random forests selected the correct marker as the top gene for every cell type except stellate cells, for which it flagged *CRISPLD2*, a gene not supported by existing literature. XGBoost, in contrast, correctly identified *COL1A1* for stellate cells but failed to select the canonical markers for acinar and ductal cells as the top-ranked features. These findings underscore the need for caution when relying on ML-based feature rankings to develop predictive marker panels, such as those implemented in recent software packages^18^.

### 4.2 Analysis of Ncx1 ^−/−^ and wild type mouse embryos

We analyzed a publicly available dataon mouse embroys generated by Azzoni et. al. (2021)^73^ to study activation trajectories at the onset of circulation. Circulation refers to the heart-driven pumping of blood, which generates mechanical forces in the embryo that are essential for triggering critical developmental processes, such as the Endothelial-to-Hematopoietic Transition (EHT). In particular, EHT is a foundational developmental process essential for life, as it is the original source of all lifelong blood and immune cells. During EHT, specialized endothelial (vessel-lining) cells transform, detach, and are ultimately released into the body as the first hematopoietic stem cells. Wild-type embryos possess a normal heartbeat and hemodynamic flow, allowing EHT to be activated through proper circulation. Ncx1^−/−^ embryos, on the other hand, do not go through a proper circulation process, which in turn leads to impaired EHT activation. Therefore, the comparison between Ncx1^−/−^ and wild-type embryos is critical for identifying the genes and functional processes that are crucial for circulation.

We obtained the gene expression data and cell type annotations from the European Bioinformatics Institute single cell expression atlas website.^74^. For our analysis, we only kept the Ncx1^−/−^ and wild-type cells. We next performed a similar filtering step as in Section 4.1 to pick differentially expressed genes and remove highly correlated genes. The final analysis-ready dataset had *N* = 444 observations and *p* = 59 genes with 244 Ncx1^−/−^ and 200 wild-type cells. CBKMR produced PIP values of 1 for 8 out of the 50 genes, rendering those genes significant for classification. Of these, *CD44* was previously reported as an essential marker^75^ for EHT. This also corroborates the authors’ finding that *CD44* is generally downregulated in the case of impaired EHT. Among other selected genes, *Sox9* was closely related to a key EHT transcription factor *Runx1* in other studies^76^. Notably, we also performed random forests and XGBoost on the filtered data. Neither method ranked these two biologically important genes among their top 10 features, indicating their limitations in complex settings. CBKMR also selected *Gja5* that was highly correlated with the arterial marker gene *Gja4* (correlation=0.7).

## 5 Conclusion

In this work, we introduce a copula-based Bayesian kernel machine regression (CBKMR), a flexible framework that separates the modeling of marginal mean structure from the kernel-induced dependence among observations. By using a Gaussian copula parameterized through an automatic relevance determination (ARD) kernel, CBKMR more reliably captures complex gene–outcome relationships that generalized BKMR (GBKMR) models based on latent Gaussian process (GP) formulations often struggle to estimate for discrete outcomes. This improvement stems from the ability of the copula construction to accommodate non-Gaussian, heterogeneous dependence patterns between observations that are not well represented by a latent GP. To enable interpretable marker discovery, we incorporate a zero-mass spike-and-slab prior on the ARD relevance parameters, yielding intuitive variable selection through posterior inclusion probabilities (PIPs). Finally, to ensure scalability in large datasets, we develop NNCBKMR, which uses nearest-neighbor GP–based sparse Cholesky factorizations^49^ to substantially reduce computational cost.

In extensive simulation studies conducted under model misspecification (data generated from a logistic GBKMR model), logistic CBKMR consistently outperforms both logistic GBKMR and leading ensemble machine-learning methods (e.g., random forests, XGBoost), underscoring its utility as a novel and effective marker-selection framework. Notably, NNCBKMR achieves accuracy comparable to the full CBKMR model while providing a multi-fold speedup. In applications to two scRNA-seq datasets—type 2 diabetes (T2D) islet cell types^69^ and mouse embryo differentiation^73^—CBKMR successfully recovers nearly all pathologist-annotated marker genes in the T2D dataset and identifies biologically essential genes that separated *Ncx*1^−/−^ from wild-type cells in the embryo dataset. Several of these markers were down-weighted or entirely missed by feature-importance rankings from random forests and XGBoost, demonstrating the improved sensitivity of CBKMR to nonlinear and jointly informative molecular effects.

This work paves the way for broader use of copula-based kernel machine regression in settings characterized by complex, non-Gaussian dependence structures. Although CBKMR is robust to correlation among predictors, as demonstrated in our real-data analyses, a current limitation lies in the assumption of independent priors on the relevance parameters. This assumption may underrepresent the shared regulatory behavior of genes within the same biological pathway, potentially motivating future extensions that incorporate structured or group-wise dependence in the prior^77^. Additionally, further scalability may be achieved by incorporating variational inference into the NNCBKMR framework^78^, allowing fast approximate inference in large-sample settings. A final and important direction is to extend CBKMR to explicitly model spatial dependence, enabling spatially informed marker detection in emerging spatial omics datasets.

### A Proof of Lemma 1

We will follow Theorem 2.1 of Schäfer et. al. (2021)^79^. for an outline of this proof. Note that, when the individual CDFs $F_{i}$’s are continuous, there is no need for discrete transformation (DT) approximation and the density of y given a copula correlation matrix $\Sigma_{r}$ (assuming that y is already permuted according to the maxmin ordering^67^) can be described as,

(A.1) $$f\left(y|\Sigma_{r}\right)=\frac{1}{{\left|\Sigma_{r}\right|}^{1/2}}exp\left(-\frac{1}{2}{\left[\Phi^{-1}(F(y))\right]}^{⊤}\left(\Sigma_{r}^{-1}-I_{n}\right)\left[\Phi^{-1}(F(y))\right]\right)\prod_{i=1}^{N}f_{i}\left(y_{i}\right)$$

Since $\Sigma_{r}$ does not contain $\beta_{0}$ and $\beta_{1}$ in $f\left(y|\Sigma_{r}\right)$ as well as in the mathematics that follows. Once the sparsity structure 𝒮 is obtained through kd-tree algorithm, the Kullback-Leibler (KL) divergence between the density of y with a copula correlation matrix $\Sigma_{r}$ and the same with a copula correlation matrix ${\left(A_{𝒮}A_{𝒮}^{⊤}\right)}^{-1}$ for a positive definite matrix $A_{𝒮}$ is given by,

(A.2) $$KL\left(f\left(y|\Sigma_{r}\right)\|f\left(y|{\left(A_{𝒮}A_{𝒮}^{⊤}\right)}^{-1}\right)\right)\;=\int log\left(\frac{f\left(y|\Sigma_{r}\right)}{f\left(y|{\left(A_{𝒮}A_{𝒮}^{⊤}\right)}^{-1}\right)}\right)f\left(y|\Sigma_{r}\right)dy\;\;=\int log\left(\frac{{\left|\Sigma_{r}\right|}^{-1/2}exp\left(-\frac{1}{2}z^{⊤}\left(\Sigma_{r}^{-1}-I_{n}\right)z\right)\prod f_{i}\left(y_{i}\right)}{{\left|A_{𝒮}A_{𝒮}^{⊤}\right|}^{1/2}exp\left(-\frac{1}{2}z^{⊤}\left(\left(A_{𝒮}A_{𝒮}^{⊤}\right)-I_{n}\right)z\right)\prod f_{i}\left(y_{i}\right)}\right)f\left(y|\Sigma_{r}\right)dy$$

where $z=\Phi^{-1}(F(y))$. The marginal densities $\prod f_{i}\left(y_{i}\right)$ and the identity matrices $I_{n}$ within the quadratic forms cancel out. By applying the transformation $z=\Phi^{-1}(F(y))$, the expression simplifies to the KL divergence between two centered multivariate Gaussian distributions:

$$KL\left(𝒩\left(0,\Sigma_{r}\right)\|𝒩\left(0,A_{𝒮}A_{𝒮}^{⊤}\right)\right)\;=\frac{1}{2}E_{z∼𝒩\left(0,\Sigma_{r}\right)}\left[log\frac{1}{\left|A_{𝒮}A_{𝒮}^{⊤}\Sigma_{r}\right|}+z^{⊤}\left(A_{𝒮}A_{𝒮}^{⊤}\right)z-z^{⊤}\Sigma_{r}^{-1}z\right]\;\;=\frac{1}{2}\left(tr\left(\left(A_{𝒮}A_{𝒮}^{⊤}\right)\Sigma_{r}\right)-N-log\left(\left|\Sigma_{r}\right|-log\left(\left|A_{𝒮}A_{𝒮}^{⊤}\right|\right)\right)\right)$$

Since $A_{𝒮}$ is sparse lower triangular matrix, we can directly argue following Theorem 2.1 of Schäfer et. al. (2021)^79^ that the KL term will be minimized when $A_{𝒮}=U_{𝒮},U_{𝒮}$ is described as in Lemma 1.

## Figures and Tables

**Figure 1:**
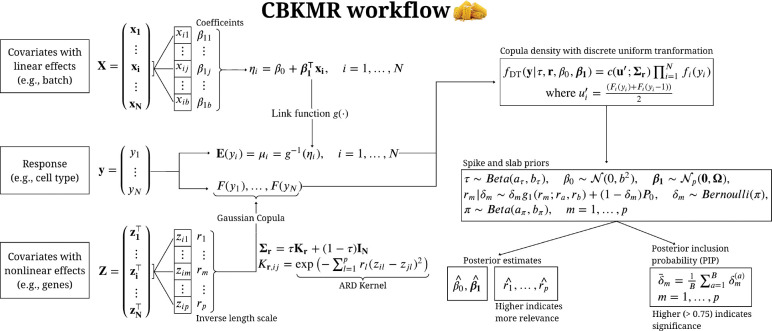
CBKMR workflow diagram with key modeling steps.

**Table 1:** Time comparison per 2,000 iterations between CBKMR and NNCBKMR (in minutes). GBKMR takes 128 minutes (2 hours) to complete 2,000 iterations for *N* = 700 and *p* = 10.

N	CBKMR		NNCBKMR (k=5)		NNCBKMR (k=10)		NNCBKMR (k=20)		NNCBKMR (k=40)	
	p = 10	p = 20	p = 10	p = 20	p = 10	p = 20	p = 10	p = 20	p = 10	p = 20
700	4.71	4.86	0.63	0.66	0.72	0.85	1.11	1.15	1.70	1.92
1,000	12.25	12.75	0.94	0.96	1.17	1.20	1.61	1.71	2.60	2.79
